# An Integrated Clinical‐MR Radiomics Model to Estimate Survival Time in Patients With Endometrial Cancer

**DOI:** 10.1002/jmri.28544

**Published:** 2022-12-09

**Authors:** Xingfeng Li, Diana Marcus, James Russell, Eric O. Aboagye, Laura Burney Ellis, Alexander Sheeka, Won‐Ho Edward Park, Nishat Bharwani, Sadaf Ghaem‐Maghami, Andrea G. Rockall

**Affiliations:** ^1^ Department of Surgery and Cancer Imperial College London UK; ^2^ Chelsea and Westminster Hospital NHS Foundation Trust London UK; ^3^ Imaging Department Imperial College Healthcare NHS Trust London UK

**Keywords:** endometrial cancer, survival analysis, radiomics, feature selection, Cox proportional hazards model, T2‐weighted MRI

## Abstract

**Background:**

Determination of survival time in women with endometrial cancer using clinical features remains imprecise. Features from MRI may improve the survival estimation allowing improved treatment planning.

**Purpose:**

To identify clinical features and imaging signatures on T2‐weighted MRI that can be used in an integrated model to estimate survival time for endometrial cancer subjects.

**Study Type:**

Retrospective.

**Population:**

Four hundred thirteen patients with endometrial cancer as training (N = 330, 66.41 ± 11.42 years) and validation (N = 83, 67.60 ± 11.89 years) data and an independent set of 82 subjects as testing data (63.26 ± 12.38 years).

**Field Strength/Sequence:**

1.5‐T and 3‐T scanners with sagittal T2‐weighted spin echo sequence.

**Assessment:**

Tumor regions were manually segmented on T2‐weighted images. Features were extracted from segmented masks, and clinical variables including age, cancer histologic grade and risk score were included in a Cox proportional hazards (CPH) model. A group least absolute shrinkage and selection operator method was implemented to determine the model from the training and validation datasets.

**Statistical Tests:**

A likelihood‐ratio test and decision curve analysis were applied to compare the models. Concordance index (CI) and area under the receiver operating characteristic curves (AUCs) were calculated to assess the model.

**Results:**

Three radiomic features (two image intensity and volume features) and two clinical variables (age and cancer grade) were selected as predictors in the integrated model. The CI was 0.797 for the clinical model (includes clinical variables only) and 0.818 for the integrated model using training and validation datasets, the associated mean AUC value was 0.805 and 0.853. Using the testing dataset, the CI was 0.792 and 0.882, significantly different and the mean AUC was 0.624 and 0.727 for the clinical model and integrated model, respectively.

**Data Conclusion:**

The proposed CPH model with radiomic signatures may serve as a tool to improve estimated survival time in women with endometrial cancer.

**Evidence Level:**

4

**Technical Efficacy:**

Stage 2

Endometrial cancer is the most common gynecological cancer, with 417,000 new cases diagnosed globally in 2020.^1^, ^2^ The 5‐year overall survival rate of endometrial cancer patients ranges from 74% to 91%.^3^, ^4^, ^5^ To study the survival time of endometrial cancer patients, survival analysis methods have been extensively applied.^4^, ^5^, ^6^ Currently utilized examples include the non‐parametric Kaplan–Meier method and the semi‐parametric Cox's proportional hazards (CPH) method.^4^, ^5^, ^7^, ^8^, ^9^, ^10^, ^11^ Based on the CPH model, it is possible to evaluate a single covariate's and/or the combination joint covariates effects on the survival time estimation. For instance, a combination of age, cancer histologic grade, socioeconomic factors, and other clinical prognostic factors have been investigated in endometrial cancer survival studies within the framework of the CPH model.^12^, ^13^, ^14^, ^15^

Until the advent of radiomics, image biomarkers have been insufficiently studied as potential survival predictors for endometrial cancer. Radiomics is a rapidly expanding field of research in oncology.^16^, ^17^ There have been studies evaluating the application of radiomics, usually based on multi‐sequence MRI features, for example Kurtosis from contrast‐enhanced T1‐weighted MRI to predict survival time in endometrial cancer, but these studies had several limitations.^17^, ^18^, ^19^ Specifically, these studies employed a dataset with less than 200 cases, thus with a small number of sample sizes and radiomic features (less than 100 features), which could lead to a large bias for the model estimation.^20^, ^21^ Moreover, as these studies did not conduct a validation using independent external testing data, there was no model validation for survival time prediction.^17^, ^18^, ^19^ Finally, these studies did not include or combine clinical prognostic variables in the CPH model for the survival time prediction.^17^, ^18^, ^19^ As a result, these previous studies have most likely not evaluated the true potential of radiomic features for survival time prediction in endometrial cancer.^17^, ^18^, ^19^

To overcome these limitations, this retrospective study aimed to identify a radiomic signature using pelvic MRI data that could estimate survival time in endometrial cancer. Furthermore, we sought to develop and validate an integrated clinical‐radiomic model that might be used to tailor adjuvant management for women based on their personalized risk features.

## Materials and Methods

This retrospective study protocol was approved by the Institutional Review Board (IRB), and the Research Ethics Committee reference number for this study is 17/LO/0173. The requirement for written informed consent was waived due to the retrospective design of this study. This retrospective study will develop and test a model which will be further validated as part of a larger prospective study (ClinicalTrials.gov NCT03543215, https://clinicaltrials.gov/).

### Training and Validation Datasets

**Figure 1:**
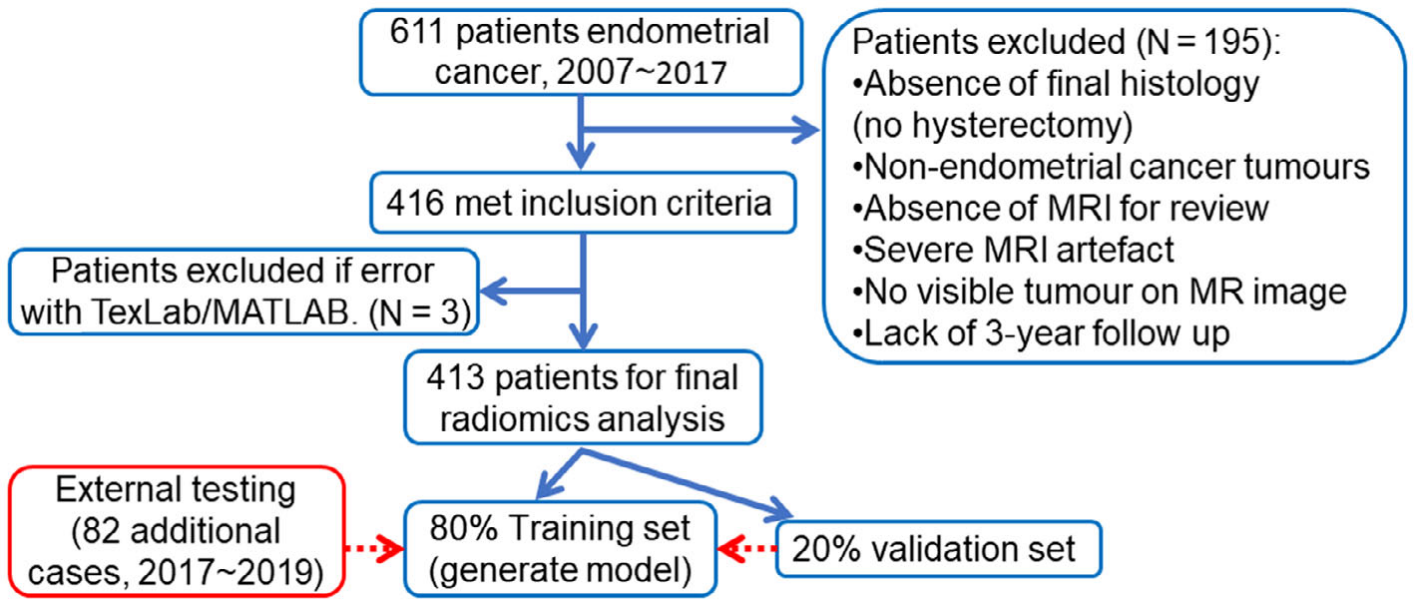
Flow chart of patient selection. After exclusion, 413 cases were included and used to generate the final model. Eighty‐two cases were used as external testing dataset.

Images were acquired between Feb 2007 and Aug 2017 (Fig. 1), and 270 of the initially considered 611 subjects were obtained from a previous study.^22^ The training and validation datasets were obtained from 15 UK hospitals and centers with different parameters and protocols (Table 1). Table 1 shows the scan parameters for collecting 411 subjects of training/validation dataset which excluded two subjects because the scan parameters information was not available. The sagittal T2‐weighted image was chosen for radiomic analysis as this was part of the standard protocol from all referral centers whereas availability of other sequences was more variable. As T2‐weighted images were included from different centers in the study, image pre‐processing and image normalization was required to minimize the difference between different scanners and sequences.

**TABLE 1:** Scan information for training/validation datasets

	GE	Philips	Siemens
MRI manufacturer (413 cases)	108	163	142

Scanner parameters (411 cases)	Mean	Median	Std
Echo time (msec)	101.81	100	16.64
Repetition time (msec)	4653.02	4100	2100.78
Slice thickness (mm)	4.31	4	0.66
Spacing between slices (mm)	4.95	5	0.55
Reconstruct matrix size	441.47	512	113.71
Magnetic field strength (T)	1.51	1.5	0.15

Std = standard deviation; msec = millisecond; mm = millimeter; T = tesla ; GE = General Electric Company, NY, USA.

Clinical data, including the patient age at diagnosis, date of surgery, type and grade of tumor, the international federation of obstetricians and gynecologists (FIGO) stage, presence of lymphovascular space invasion, and any adjuvant or neoadjuvant treatment of these subjects were obtained from an online medical records system.^23^ Survival time was defined as the time from the date of surgery until the date of death, with final censor date on August 3, 2020.

**Figure 2:**
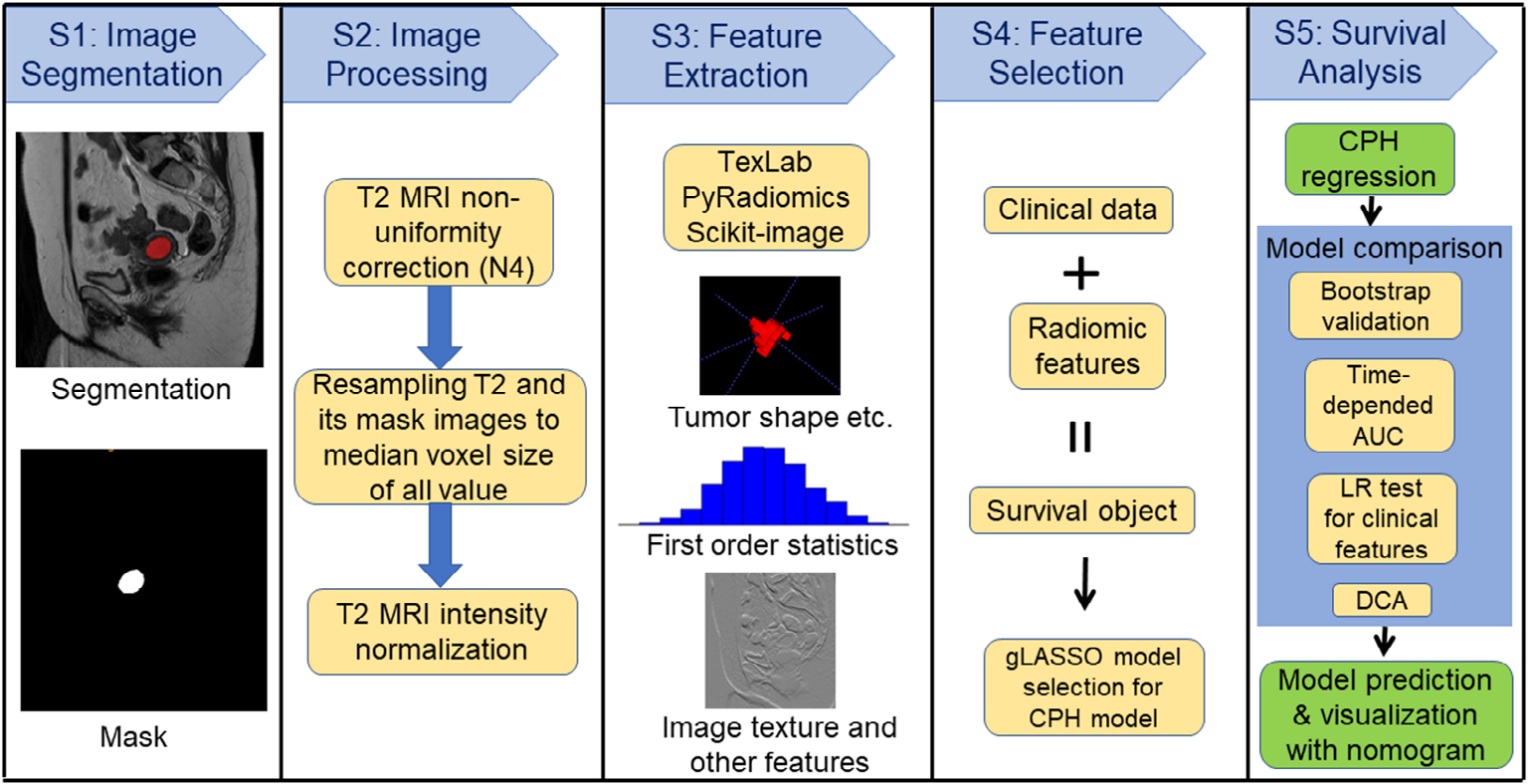
Pipeline for the study. Five steps were included as shown in the column. LR = likelihood‐ratio test; gLASSO = group Least Absolute Shrinkage and Selection Operator; CPH = Cox proportional hazards model; DCA = decision curve analysis; AUC = area under the receiver operating characteristic (ROC) curve.

The inclusion criteria regarding MRI were as follows: 1) no severe motion artifacts in T2‐weighted images that obscured the tumor mass, as determined by radiologists subjectively, 2) sufficient size of the tumor on images (i.e., the tumor could be identified on more than one MRI slice before image resampling), and 3) the T2‐weighted sequence passed the image pre‐processing steps (see step two in Fig. 2). The inclusion criteria regarding clinical data were: 1) availability of censoring or noncensoring survival information, information on lymphovascular space invasion, histological risk, and histological type, 2) availability of age at diagnosis and surgery date, 3) no other type of co‐existing cancer. After exclusion of patients based on image and clinical criteria, 413 cases were used in this study (Table 2). The ratio for splitting the training and validation was 80:20 (N = 330 for the training data; N = 83 for the validation data) with balance the survival object (i.e., the combination of time and death information) distributions within the splits.

**TABLE 2:** Training (including validation) and testing patient demographics

Clinical Information	N (training)	% (training)	N (testing)	% (testing)	*P* value
Age: mean (SD)	66.64 ± 11.5		63.25 ± 12.4		0.024
Under 50	29	7	11	13.4	0.79
50–59	78	18.9	24	29.3	0.55
60–69	133	32.2	15	18.3	0.18
70 and older	173	41.9	32	39.0	0.18
Histological type					0.0011
Endometrioid	304	73.6	69	84.1	
Carcinosarcoma	44	10.7	1	1.2	
Serous	39	9.4	4	4.9	
Clear cell	18	4.4	2	2.4	
Mixed high grade	7	1.7	2	1.7	
Undifferentiated	1	0.2	3	3.7	
NET small cell			1	1.2	
Grade					3.72 e‐04
1 (low grade)	124	30.0	43	52.4	
2 (intermediate grade)	130	31.5	20	24.4	
3 (high grade)	159	38.5	19	23.2	
Overall FIGO stage					0.1738
Stage I	292	70.7	59	72	
IA	199	48.2	45	54.9	
IB	93	22.5	14	17.1	
Stage II	31	7.5	5	6.1	
Stage III	64	15.5	7	8.5	
IIIA	18	4.4	4	4.9	
IIIB	6	1.4	0	0	
IIIC	40	9.7	3	3.7	
Stage IV	25	6.1	1	1.2	
IVA	18	4.4	0	0	
IVB	7	1.7	1	1.2	
Other (missing)	1	0.2	1	1.2	
Clinical risk score					0.0388
Low	150	36.3	41	50	
Intermediate	78	18.9	15	18.3	
High	96	23.2	9	11.0	
Advanced	89	21.5	16	19.5	
Unknown			1	1.2	
Censored					0.0096
Censoring	317	76.8	74	90.2	
Death	96	23.2	8	9.8	

N = 413 (training/validation), N = 82 (testing). SD = standard deviation; NET = neuroendocrine tumor; FIGO = The International Federation of Gynecology and Obstetrics. Staging version.

### Testing Dataset

**Figure 3:**
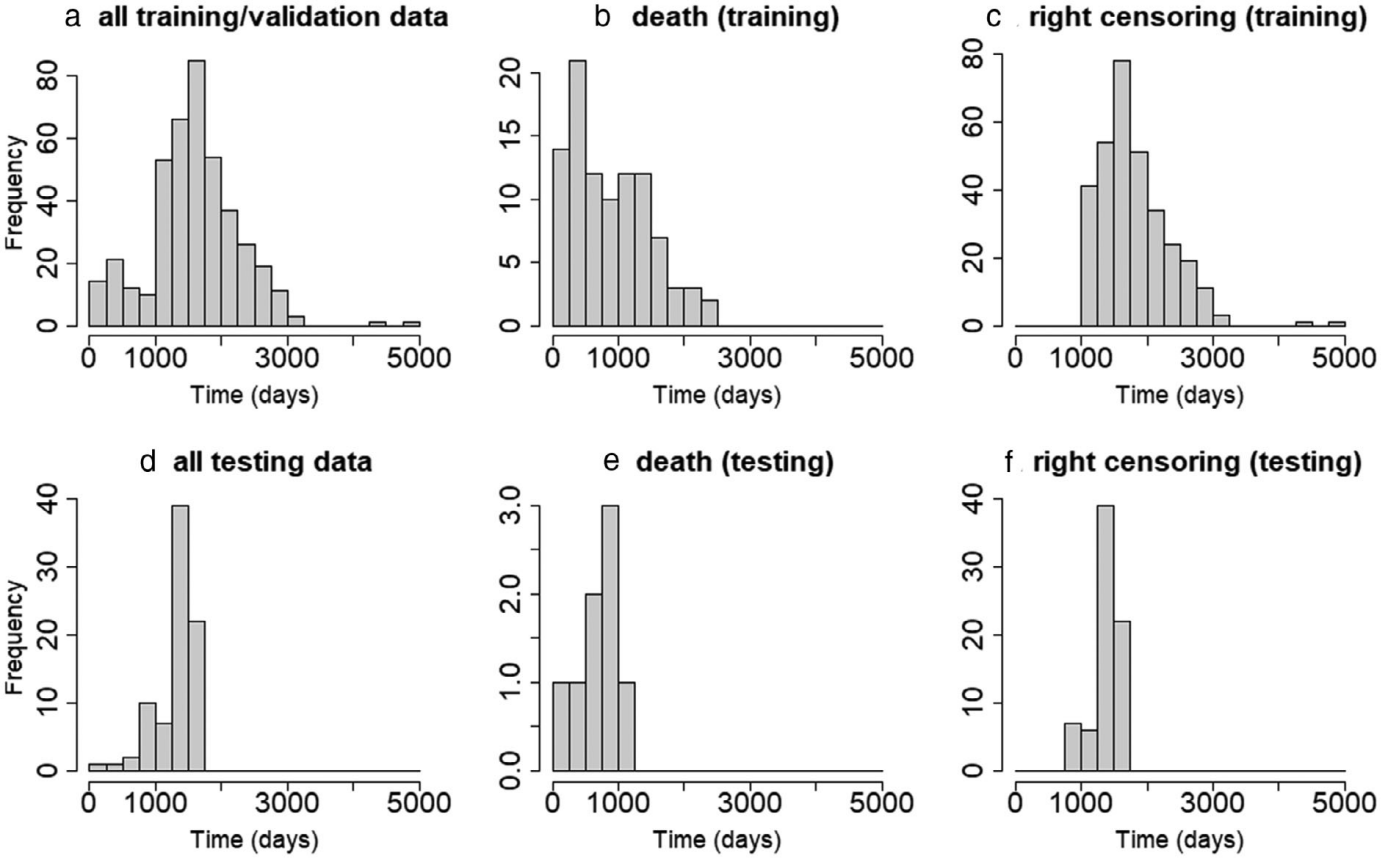
Histograms of the survival data for all training/validation **(a)** and testing **(d)** datasets and histograms of the censoring and the noncensoring datasets. The noncensoring data **(b)** and the right censoring data **(c)** distributions from the training dataset. The noncensoring data **(e)** and the right censoring data **(f)** distributions from the testing dataset.

Overall, 82 additional patients from three hospitals in the UK with endometrial cancer were included in the testing dataset, the scans being acquired between May 2017 and July 2019 (Table 2). For the testing dataset, the beginning time was the surgery date also, of which the earliest was in May 2017, and the ending time was in July 2019, and the close of the study was on December 1, 2021. 74 of the 82 cases were right censoring; at the close of the study on December 1, 2021, eight patients had died and 74 patients had survived. The right censored survival times underestimate the true (but unknown) time to event/death.^6^ The distribution of the training and testing datasets are displayed in Fig. 3.

### Radiomics Study Pipeline

Figure 2 shows the radiomics study pipeline for the survival analysis. There were five steps in this pipeline. The first and second steps were designed to analyze images, including manual image segmentation (prior to image re‐sampling), MRI nonuniformity correction, image resampling, and image normalization. Specifically, Digital Imaging and Communications in Medicine file formats were downloaded from the picture archiving and communication systems, de‐identified and converted to the simpler Neuroimaging Informatics Technology Initiative (NIFTI) format.

An interactive tool (ITK‐snap, version 3.6.0, http://www.itksnap.org) for semi‐automatic segmentation of sagittal orientation T2‐weighted MRI was employed for manual slice‐by‐slice tumor segmentation by two radiologists in‐training (JR, 5 years, with assistance from AS, 3 years).^24^ After loading the T2‐weighted MRI, the paintbrush tool was used to label all voxels containing visible tumor on each sagittal slice. Once all slices containing tumor had been labeled, the segmentation mask was saved as NIFTI format for pre‐processing steps. This process was repeated for T2‐weighted MRI in every image set. This was then checked by two radiology consultants (AR, 19 years' experience and NB 15 years' experience), who corrected the segmented tumor masks, without further went through all cases together again. The radiologists were blinded to the outcome measures. One example of the image segmentation is displayed in step 1 of Fig. 2.

The T2‐weighted images were pre‐processed according to step two as shown in Fig. 2. First, all image voxel sizes were obtained from NIFTI files with T2‐weighted MRI header files, and the median voxel size of all data was calculated. The image reconstruction matrix size in sagittal orientation was between 256 and 864 (Table 1). The median resolution (image voxel size) of all T2‐weighted images (including both training/validation and testing datasets) was 0.625 mm × 0.625 mm × 5 mm. Then, T2‐weighted images were processed using an N4 toolbox for MRI nonuniformity bias correction, and to remove artifacts due to the inhomogeneity of magnetic fields (https://github.com/ANTsX/ANTs/wiki/N4BiasFieldCorrection).^25^ Following bias correction, T2‐weighted MRI and its masks were resampled to median voxel resolution (Fig. 2). For T2‐weighted MRI resampling, the cubic spline interpolation method was adopted. For segmented tumor masks (binary image), a nearest neighbor interpolation method was used for image resampling. Next, the intensity of resampled T2‐weighted images was normalized using the following equation:

(1) $$I_{\mathrm{normalized}}=100\frac{I-\bar{I}}{\mathrm{std}\left(I\right)},$$

where I is image intensity, $\bar{I}$ is the mean value of the image intensity within the volume, and *std* is the standard deviation of the image volume. Finally, the TexLAB tool (version 2.0) on MATLAB (version R2019a; The MathWorks Inc., Natick, MA, USA; http://www.mathworks.com/), PyRadiomics (version 3.0.1, https://github.com/AIM-Harvard/pyradiomics), and Scikit‐image (version 0.19.2, https://scikit-image.org/), both implemented in Python (Python Software Foundation, version, Python3.8, https://www.python.org/) were used to perform feature extraction as shown in Fig. 2.^26^, ^27^ After elimination of identical features by a correlation method, in total 958 radiomics features were extracted from T2‐weighted MRI and its associated segmentation masks. T2‐weighted MRI was included because image intensity‐based features were derived from T2‐weighted MRI images. Endometrial cancer tumor region was the only region of interest in this study.

### Feature Selection

The fourth step was to select features for survival analysis. Radiomic and clinical feature selections were performed within the framework of statistical model selection, and the CPH model was used to study the relationship between predictor variables and survival time. In the CPH model, the time and event/death were treated as dependent variables (survival object); 958 radiomics features, cancer risk score (which includes FIGO stage), cancer grade, and age were included as predictors (independent variables) for model selection.^9^ Cancer risk score and grade were defined according to FIGO.^23^, ^28^ Before applying the model selection method, all 959 features (958 MRI features + age) were normalized using a Z‐score method (similar to Eq. 1, except multiply 100). To avoid model overfitting, a 10‐fold cross validation for penalized Cox regression models with grouped covariates was adopted to determine the optimal regularization parameter lambda (*λ*). Specifically, a group exponential least absolute shrinkage and selection operator (gLASSO) was used to select statistical models.^29^ The maximum iteration of the 10‐fold cross validation was set to be 1 million times in the model fitting. The final selected CPH model was then applied to calculate the survival time.

### Statistical Analysis

The R software (version 4.0.2; R Foundation for Statistical Computing, Vienna, Austria; http://www.R-project.org) was used for statistical analysis. Model selection package “grpreg” (version 3.4.0, https://cran.r-project.org/web/packages/grpreg/index.html) was applied to determine the optimal CPH model. The criteria for the optimal model were model simplicity and accuracy (i.e., minimize the combination of the L1 and L2 norm).^29^, ^35^ The “Survival” package (version 3.4.0, https://cran.r-project.org/web/packages/survival/index.html) was used to implement CPH model. A bootstrap resampling method was developed to assess the predictive performance of the CPH model using a Score() function from a “riskRegression” R library (version 2021.10.10, https://cran.r-project.org/web/packages/riskRegression/index.html). Nomograms were generated using a “regplot” R package (version 1.1, https://cran.r-project.org/web/packages/regplot/index.html).

Survival analysis was implemented based on the selected integrated model as shown in step five of Fig. 2. The gLASSO method produced model selection results with randomness. The most common output by the gLASSO method was adopted. Once the survival time prediction according to the CPH model was established with the gLASSO method, a nomogram was created as a graphical representation of the integrated model. The nomogram was applied to visualize the prediction survival probability. To study the influence of the radiomic features on the survival probability, two models were constructed and compared for the estimation. The first model was based on clinical information only; the predictors of the model included only age and cancer grade. The risk score was not included in the final model as it had not been selected by the gLASSO method, which may because the cancer grade and risk score are correlated. The second model used both clinical information (age and cancer grade) and three radiomic features selected by the gLASSO method.

Additional analyses were performed to validate the model based on the prediction using the “riskRegression” library. The time‐dependent area under the receiver operating characteristic (ROC) curve (AUC) was calculated from the validation (AUC is specified for AUC of ROC in this study). For the model validation, 80% of the 413 cases were used to generate the CPH model, while the rest of the datasets were employed to validate the predictive performance. Using a stratified sampling method, the training and validation datasets were split with survival objects (time/death). To validate the CPH model, the bootstrap resampling method with a sample size of 10 at each time point was adopted. Because the bootstrap method and stratified sampling method have randomness, and as a typical example, the AUC was calculated and displayed because AUC is widely used criterion for measure discrimination. To reduce the effect of the randomness in the evaluation study by using bootstrap method, the concordance index (CI), which measures the prediction accuracy, was calculated with 10 repetitions (with different training validation datasets splits). The time point started at 100 days and terminated at 1825 days with a 5‐day interval. Similarly, additional external testing cases were used to test the CPH model prediction performance.

To study the effect of the radiomic features and clinical variables on survival time estimation, decision curve analysis (DCA) was applied to evaluate the clinical, radiomic, and integrated models for net benefit.^30^ Net benefit is calculated for each possible threshold probability which puts benefits and harms on the same scale. Threshold probability is the expected benefit of treatment is equal to the expected benefit of avoiding treatment.^30^ By varying the threshold probability, DCA allows us to examine whether one model is superior to another at a certain range of threshold probability with respect to the net benefit.

A likelihood‐ratio test method was applied to study the importance of the radiomic and clinical features for survival time prediction. Furthermore, training and test datasets were compared using Chi‐squared tests for categorical data and two‐sample *t*‐tests for continuous data (Table 2). A *P*‐value <0.05 was considered statistically significant.

A diagnostic analysis was carried out to study the feature variation obtained from different types of scanners using 413 training/validation cases. It is not obvious to inspect the features difference from two dimensions, for example, in an image with 413 rows and 958 MRI feature columns. Therefore, dimension reduction method was applied to obtain the major components of the features from each type of scanner. Specifically, principal component analysis (PCA) was applied to study the effect of feature difference from different scanners. All features were normalized using the Z‐score method, and then a PCA was employed to split the feature dataset into different components. Four principal components were used to compare the feature variations from different scanners. Three different manufacturers GE: 108 cases, Philips: 163 cases, and Siemens: 142 cases were used to acquire sagittal T2‐weighted MRI (Table 1). Feature matrix from these three different scanners were decomposed into four components. Visual comparison was carried out to evaluate the distribution of the feature components from different scanners.

## Results

### Training and Testing Dataset Demographics

Clinical‐pathological characteristics of the patients are shown in Table 2. In addition, Fig. 3 plots the histograms of the testing dataset and a two‐sample *t*‐test that was applied to compare the training (including validation) and external testing datasets. Except for the survival time (Fig. 3b,e), all comparisons between training and testing datasets were significant. For the survival time (Fig. 3b,e), no significant differences were revealed (training dataset: 870.6 ± 592.1 days, testing dataset: 637.1 ± 314.2, *P* = 0.09). Table 2 also includes the demographic information from the testing dataset. The age at diagnosis of the testing dataset was significantly different from the training dataset (66.64 ± 11.51 years vs. 63.26 ± 12.38 years, Table 2).

### Feature Selection Results

**Figure 4:**
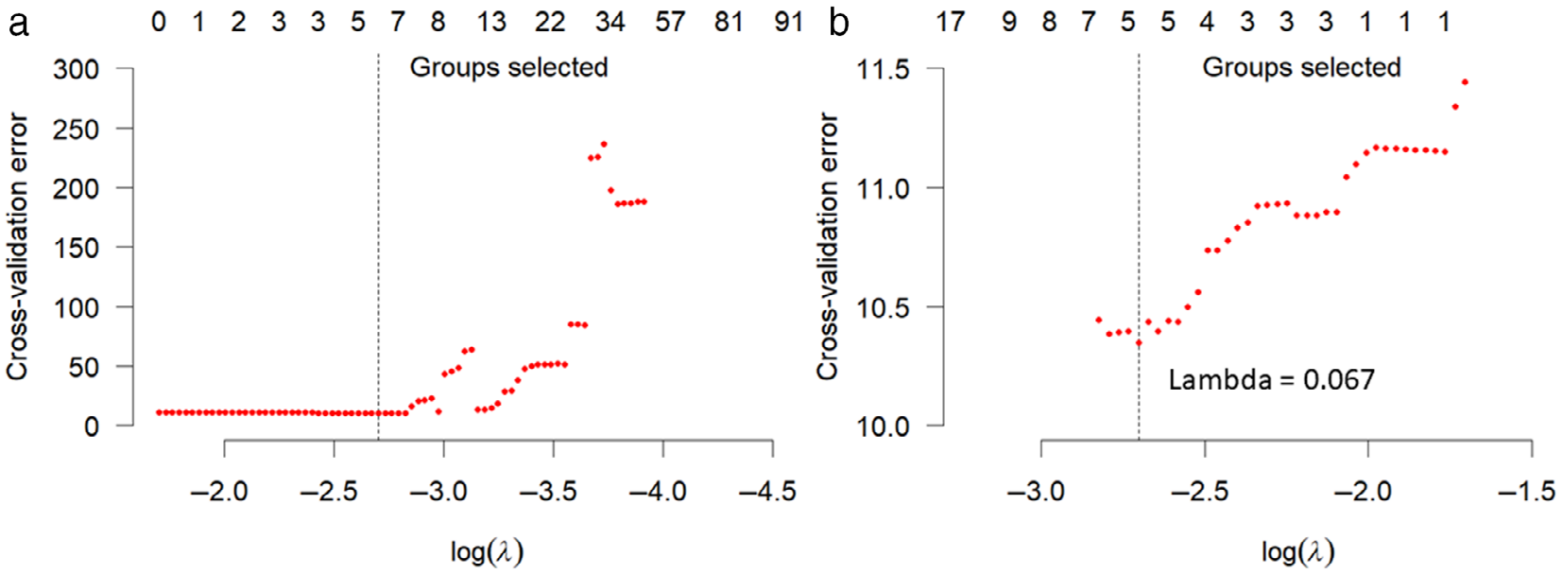
Group least absolute shrinkage and selection operator (LASSO) for feature selection. **(a)** 10‐fold cross‐validated error rates for the model selection. **(b)** Amplified version of Fig. 4a at the optimal lambda value. The vertical dotted lines indicate the minimum error, and the top of the plot gives the size of each model. Each red dot represents a *λ* value along the path. In the group LASSO method, the cross‐validation method was applied to select the tuning parameter (*λ*). Dotted vertical lines were drawn at the optimal *λ* values by using the minimum criteria (i.e., cross‐validation error). A Lambda value of 0.067 (log(*λ*) = −2.7) according to the 10‐fold cross‐validation method was computed.

Figure 4 shows the gLASSO coefficient profiles selected from 961 features. Specifically, Fig. 4a plots the 10‐fold cross‐validated error rates, and Fig. 4b shows the amplified version of the gLASSO selection plot. Five features were selected from 961 predictors and were included in the integrated CPH model. They were tumor mask minor axis radius (minorAxisRad), gray level size zone matrix (GLSZM), first order statistics (FOS), patient age at diagnosis (Age), and cancer grade (Grade). Tumor minor axis radius reflects the size of the tumor indirectly; the FOS here is the coefficient of variation, which is defined as the ratio of the standard deviation to the mean, and these values were computed within the tumor mask. This was computed after the normalized T2‐weighted image were filtered with low, low, and high wavelet filters in *x*, *y*, and *z* direction of the 3D image subsequently. The GLSZM was calculated after the normalized MRI image was converted into 25 Hounsfield unit gray level, then the large zone low gray level emphasis was computed within the tumor mask. The FOS and GLSZM represent image statistical property and intensity character. These five selected features were refit into a CPH model without the normalization of the age covariate, for the purpose of displaying in the nomogram. The survival prediction was then estimated based on the refit CPH model. The final integrated model was:

$$\text{Surv}\left(\text{Time},\text{Death}\right)=0.0548^{*}\mathrm{Age}+0.0025^{*}\text{Grade2}+1.684^{*}\text{Grade3}+0.495^{*}\text{minorAxisRad}-0.263^{*}\text{GLSZM}-0.179^{*}\mathrm{FOS}.$$

The corresponding clinical model (excluding radiomic features) was:

$$\text{Surv}\left(\text{Time},\text{Death}\right)=0.0455^{*}\mathrm{Age}+1.881^{*}\text{Grade2}+2.107^{*}\text{Grade3},$$

where Surv is the survival object, defined as a response variable in the CPH model and age was not normalized. Time is the time (number of days) from the date of surgery to the end of the study for the right censoring data. If the subject has died before the end of the study, Time is the number of days from the surgery date to the death date. Death is a status binary variable, with 1 to represent death of the subject, and 0 to denote the survival of the subject at the close of the study. Grade2 and Grade3 are the numerical variables to represent cancer grade 2 and grade 3 which were converted from cancer grade categorical variable. minorAxisRad is the tumor minor axis radius which was calculated from the tumor mask image.

### Model Training and Validation

**Figure 5:**
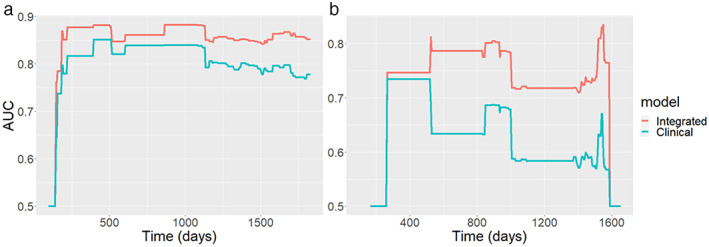
**(a)** Time‐dependent AUC summary at evaluation time points from the training/validation dataset; the AUC values are within the range of 0.5 and 0.9. The AUC for the integrated model (red curve) is consistently larger than the AUC obtained from using clinical model (green curve). **(b)** AUC obtained from the testing data; AUC values are within the range between 0.5 and 0.85.

Figure 5a plots the time‐dependent AUC based on training and validation datasets. For the clinical model, the AUC accuracy was below 80% for the time points after 1250 days, suggesting that this model is less accurate for long‐time estimation. In Fig. 5a, the integrated model had a larger AUC than the clinical model for all time points, suggesting that the integrated clinical‐radiomic model is superior to the clinical model for the prediction based on the external testing dataset for all time points (integrated model AUC: 0.853 ± 0.06, clinical model AUC: 0.805 ± 0.058). The results also showed that the CI value was significantly higher using the integrated model based on these 413 cases (integrated model CI: 0.825 ± 0.010, clinical model CI: 0.806 ± 0.011).

**Figure 6:**
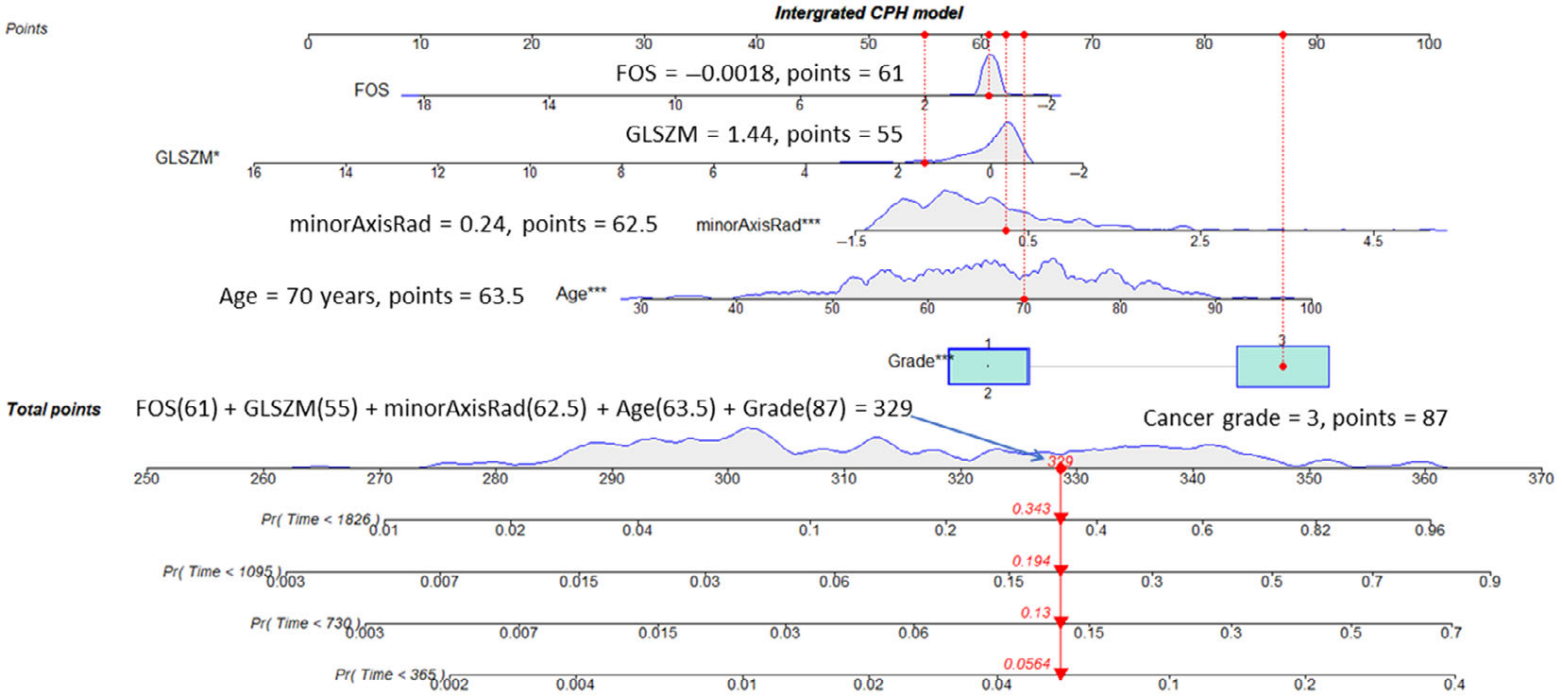
Nomogram visualization for the survival time prediction. At the top of the nomogram, a point scale was included. Beneath the scale, three radiomic features, age, and the clinical cancer grade were displayed. The refitted CPH model was adopted to predict the survival probability for 1 (365 days), 2 (730 days), 3 (1095 days), and 5 (1826 days) year periods as shown at the bottom of Fig. 6. The dotted red vertical line in the figure indicates one example of observation with an age of 70, cancer grade of 3, minor axis radius of 0.24, GLSZM of 1.44, and FOS of −0.0018. The aggregate score for this case is 329 as indicated by the red arrow vertical line at the bottom of the figure. The corresponding probability to the survival for the 5‐, 3‐, 2‐, and 1‐year periods is 0.657 (1–0.343), 0.806 (1–0.194), 0.87 (1–0.13), and 0.944 (1–0.056), respectively. GLSZM = gray level size zone; FOS = first order statistics; CPH = Cox proportional hazards.

Similarly, AUC curves were computed using the trained model on the testing dataset and the results are presented in Fig. 5b. Comparing Fig. 5a with Fig. 5b, the AUC obtained from the testing dataset is smaller than the AUC computed from the training dataset (integrated model AUC: 0.727 ± 0.085, clinical model AUC: 0.624 ± 0.070). This is because the survival time and age from the testing data were significantly different from the training and validation datasets (training and validation data: 1583.4 ± 669.6 days, testing data: 1318.7 ± 306.4 days). A likelihood‐ratio test showed a significant difference between the integrated model and clinical model based on both training and testing datasets. The CI was 0.797 for the clinical model and 0.818 for the integrated model. Based on the selected model from training data, the nomogram display the 1‐, 2‐, 3‐, and 5‐year survival probabilities is shown in Fig. 6.

The difference between the clinical model and the integrated model is small in terms of CI using the training and validation datasets, however, for the independent testing data, the CI was 0.792 for the model with age and clinical cancer grades, and the index was 0.882 for the integrated model, thus showing a significant difference (likelihood‐ratio *χ* ^2^ = 12.677). This suggests that the integrated model is robust to the different distributions of the data because age (Table 2) is statistically significant difference between the internal (training/validation) data and external dataset.

### Decision Curve Analysis

**Figure 7:**
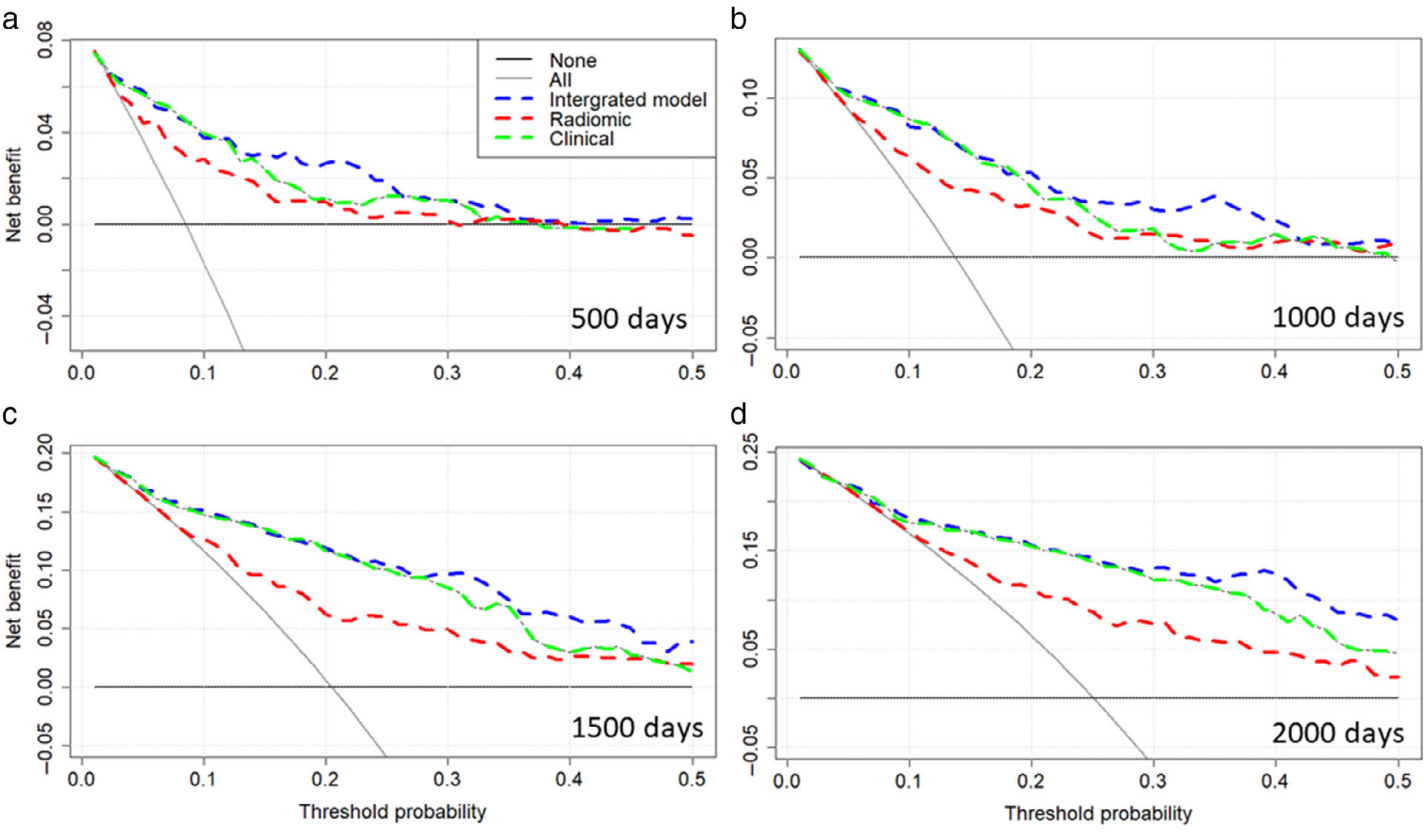
Decision curve analysis at 500 **(a)**, 1000 **(b)**, 1500 **(c)**, and 2000 **(d)** days. The net benefit is plotted against the threshold probability. If the curve is closer to the right top corner, then the corresponding model is better as it has larger net benefit. The “all” curve shows the net benefit by treating all patients, while the “none” curve denotes net benefit for treating no patients.

To study the contribution of radiomic features to survival time estimation within the CHP model, a DCA was applied to compare radiomic (includes three radiomic features only), clinical, and integrated models at 500, 1000, 1500, and 2000 days (Fig. 7). The integrated model was almost consistently on the top of other curves in Fig. 7, suggesting that the model has more net benefit than the other models for survival time prediction. The radiomic model had a larger net benefit than the clinical model when the threshold was below 0.5 (Fig. 7b,c). For a larger threshold probability (>0.45), radiomic, clinical, and integrated models had similar net benefit for a short time range estimation (Fig. 7a,b). However, for the long‐time range (2000 days or more) survival time estimation, the integrated model had a larger net benefit; comparing Fig. 7a with Fig. 7d, the gap between the curves is larger in Fig. 7d, suggesting a larger net benefit for estimations longer than 2000 days.

### Features From Different Scanners

**Figure 8:**
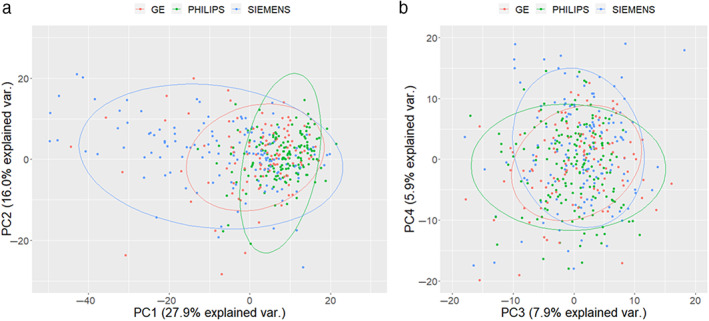
Scanner difference study. Principal component analysis for radiomics features from a different scanner. **(a)** First principal component (PC1) vs. second principal component (PC2); a relatively larger variation was observed using the Siemens scanner. **(b)** Third principal component (PC3) and fourth principal component (PC4) explain smaller percentages of the total variation, and the three different scanners show good agreement. The dots in the figure represent samples; the colors represent groups (scanner types); and the legends have three groups at the top. The ellipse represents the core area added by the default confidence interval of 68%, which facilitates the separation between the observation groups. No clear separation of the sample based on the three MRI vendors was observed. var. = variance; PC = principal component.

From PCA analysis, features components from different scanners were overlaid onto each other in Fig. 8. Similarities in the feature distribution was observed, although as shown in Fig. 8a, radiomic features from the Siemens scanners had a larger variation. For the 3rd and 4th principal components (PC3/4) (Fig. 8b), the distribution of the radiomic features obtained from different scanners were smaller, suggesting good agreement for the features from different scanners.

## Discussion

We have developed the CPH model using features from sagittal T2‐weighted MRI and clinical variables for survival time estimation based on gLASSO method. We studied the effect of the radiomic features within the model and found radiomic features from MRI are useful biomarkers to predict survival time in patients with endometrial cancer.

We identified a set of radiomic signatures using pelvic MRI that could potentially aid in accurately estimating survival time for patients with endometrial cancer. In combination with clinical features, our integrated radiomics model outperformed the clinical model in predicting survival time. We validated and compared the integrated and clinical models using both internal (training and validation) and independent external (testing) datasets. Furthermore, the CPH model with a nomogram for visualization provided a graphical, straightforward, and noninvasive method of predicted survival, which could be used in clinical settings and therefore has potential to facilitate personalized medicine. The multiple centers and scan machines used in this study presented challenges for model building, but this setup also implies that the findings are likely to be generalizable. Multiple modeling techniques were evaluated, and feature selection was utilized to avoid overfitting of the model. The radiomics quality score which determines the validity and completeness of radiomics studies, and transparent reporting of a multivariable prediction model for individual prognosis or diagnosis (TRIPOD) guidelines were adhered to ensure quality of both scientific methods and reporting.^31^

In contrast to quantitative MRI such as apparent diffusion coefficient (ADC) from diffusion‐weighted imaging, T2‐weighted MRI signal depends on many variable factors including the acquisition protocol, the coil profile, the scanner type, and therefore it is not the standard method to normalize the image intensity for cross‐subjects comparison. We adopted Z‐score‐like method to normalize the image intensity, other methods such as min/max normalization or scaling the image intensity to common max value can also be used. An alternative method to reduce the image intensity difference of T2‐weighted images acquired from different centers and scanners is to normalize to a reference tissue outside the tumor‐affected region such as cerebrospinal fluid in brain or bladder where baseline water signal can be obtained. Although the image intensity features such as mean intensity value within the tumor mask will be affected by different image normalization steps, the tumor shape, volume, and image complexity radiomic features will not be affected by the image normalization step.

Most of the published studies have focused on addressing the classification problem in endometrial cancer using radiomics.^17^, ^18^, ^19^ Studies have applied this radiomic technology to endometrial cancer survival prediction models.^32^, ^33^ Fasmer et al developed an MRI‐based whole‐volume tumor radiomic signature for the prediction of high‐risk features.^19^ Radiomic features were studied to predict poor progression‐free survival.^19^ Meanwhile, Ytre‐Hauge et al applied radiomics to study survival in women with endometrial cancer.^17^ They reported that high kurtosis in contrast‐enhanced T1‐weighted MRI predicted reduced recurrence and progression‐free survival (hazard ratio 1.5), but their study used only 180 patients without model validation,^17^ compared with 495 cases in this study. Furthermore, they used contrast‐enhanced T1‐weighted MRI images.^17^ However, we obtained the features from T2‐weighted image, which is a sequence that clearly delineates most endometrial cancers without the use of gadolinium and the sagittal T2‐weighted sequence is the mainstay of MRI protocols for staging endometrial cancer, enabling the development of a generalizable tool. We found that tumor size reflected by minor axis radius was an important biomarker for survival time estimation. The minor axis radius describes the radius of the minor axis of the ellipse that reflects the tumor region indirectly.^27^ For the features of GLSZM and FOS, both features are related to the distribution of image intensities. In this study, the GLSZM was based on the image converted from Hounsfield unit, this could be due to the relationship between MRI intensity and Hounsfield unit values.^34^

In addition to using CI, we adopted multiple criteria to evaluate the models. We have applied likelihood‐ratio test, AUC, and DCA methods to compare different models. By considering the clinical utility of the specific model, DCA overcomes the limitations of traditional metrics such as AUC which only measures the diagnostic accuracy of the model.

The pipeline of this study (Fig. 2) can be extended to other malignancies for survival analysis based on integrated features. In this method, the sources of error can come from the first three steps: image segmentation, image processing, and feature extraction. For example, in the image segmentation step, error can be generated if the tumor mask is not parcellated properly. For the image processing step, the image interpolation method can introduce numerical error. In the feature extraction step, bias can be produced if only a fraction of image features is extracted from the image.

Finally, the clinical application of the nomograms could be in patient management or prioritization; as the survival time of the patient is known from the model estimation, so treatment for patients could be arranged in a more efficient way. The integrated radiomics model may also enable better stratification of patients enrolling into clinical trials, as it has higher AUC and CI value than the model with only clinical variables.

### Limitations

This was a retrospective study and therefore, there was a risk of bias and missing data. The study also only included patients who had undergone surgery and had an MRI with paired clinical data available. While the model was assessed based on the external testing dataset, there was slight variation in demographics when directly comparing the training and validation datasets. In the testing dataset, there were less women in the older 59‐to‐70‐year group and more women with endometrioid low grade cases, namely more patients with low‐risk scores. Debatably, this would infer that the testing dataset group would be more likely to have better survival. As radiomics models perform better with more homogenous datasets such as that generated by the low‐risk cases, this may explain the slightly better performance with the testing dataset. Second, although we had also tested the composite minimax concave penalty method for the model selection in the CPH model (which produced the same model selection results as the group exponential LASSO method), other methods such as the regular elastic net and ridge models method, which may produce better results, have not been investigated in this study.^35^, ^36^ Third, survival outcomes do not only represent the effect of the disease itself, but also of patient factors (such as age or co‐morbidities) and treatment factors (such as whether the patient underwent neoadjuvant radiotherapy or chemotherapy). Study has shown that adjuvant treatment predictably improves survival for high‐risk patients.^37^ Regional and national differences in patient demographics along with treatment options offered and delivered can also impact survival disparities. This study did not consider co‐morbidities, which are likely to have a relevant impact on survival. Finally, although we normalized the images to minimize the T2‐weighted image differences obtained from different protocols, more work is needed to study effects on features for radiomics studies.

This study employed only T2‐weighted MRI data; future work could evaluate the use of additional MRI sequences or quantitative MRI such as diffusion‐weighted images with ADC maps, as well as dynamic contrast‐enhanced MRI (DCE‐MRI).^38^ Also, a possible method to explore in the future would be the boosting method or the deep survival model for the study of survival because these methods do not require the proportional hazards assumption.^39^, ^40^

## Conclusion

The integrated radiomic model and the nomogram may enable us to estimate of survival with a high degree of accuracy. Furthermore, we found that the integrated model is robust; it retained a high level of accuracy despite the variability of the independent external testing dataset, as the AUC value showed only a marginal decrease when applied to the testing dataset, in comparison to the clinical model, in which the AUC decreased markedly.
